# Novel sources of resistance to fusarium wilt in *Luffa* species

**DOI:** 10.3389/fpls.2023.1116006

**Published:** 2023-06-09

**Authors:** Sumant Bindal, Zong-ming Sheu, Lawrence Kenyon, Dalia Taher, Mohamed Rakha

**Affiliations:** ^1^ Breeding Unit, World Vegetable Center, Shanhua, Tainan, Taiwan; ^2^ Research and Development Department, R. K. Seed Farm Company, Azadpur, India; ^3^ Plant Pathology Unit, World Vegetable Center, Shanhua, Tainan, Taiwan; ^4^ Vegetable Crops Research Department, Horticultural Research Institute, Agriculture Research Center, Giza, Egypt; ^5^ Horticulture Department, Faculty of Agriculture, University of Kafr El-Sheikh, Kafr El-Sheikh, Egypt

**Keywords:** biotic stress, breeding, cucurbits, Fusarium, ridge gourd, rootstocks

## Abstract

Fusarium wilt is a serious disease of cucurbit crops including cultivated Luffa species (*Luffa aegyptiaca, Luffa acutangula*) causing considerable amount of reduction in yield and quality. Luffa is starting to be used as rootstocks for major commercial cucurbit crops, but little is known of its resistance against soilborne diseases. Here, 63 Luffa accessions from the World Vegetable Center genebank were evaluated for resistance to an aggressive isolate of *Fusarium oxysporum* f. *FoCu-1* (*Fsp-66*). According to visual screening based on disease severity rating, 14 accessions exhibited a high level of resistance against *Fsp-66*. These accessions were further evaluated for resistance against *Fsp-66* and two more isolates *FoCu-1* (isolated from infected cucumber plants) and *FoM-6* (isolated from infected bitter gourd plants). Of the 14 accessions, 11 were confirmed resistant against isolate *Fsp-66*. In addition, 13 accessions showed high resistance against isolates *FoCu-1* and *FoM-6*. This is the first report of Fusarium wilt resistance in Luffa and these sources will be valuable for the development of Luffa rootstocks/cultivars resistant to soil-borne pathogen to manage this serious disease.

## 1 Introduction

Fusarium wilt is a serious soil-borne fungal disease which causes serious losses in a wide range of vegetable crops including horticultural crops around the globe (Okungbowa and Shittu, 2012; Edel-Hermann and LeComte, 2019). Many Fusarium species are plant pathogens, but *F. oxysporum* is the most ubiquitous and well-known species (Okungbowa and Shittu, 2012). *Fusarium oxysporum* comprises several different *formae speciales* which are similar in morphology but have different host preferences, for example *F. oxysporum* f. sp. *cucumerinum*, *F. oxysporum* f. sp. *luffae*, and *F. oxysporum* f. sp. *melonis*, some ofthe types responsible for causing vascular wilt diseases in the Cucurbitaceae family, such as cucumber, watermelon, bitter gourd and muskmelon (Egel and Martyn, 2007). Although host specificity is exhibited by the different *formae speciales* of *F*. *oxysporum*, some cross pathogenicity has also been observed within the Cucurbitaceae, for example bitter gourd scions grafted on luffa rootstocks recently have shown wilting in Taiwan (Williams, 1996). Similarly, a cucumber isolate of *F*. *oxysporum* has been identified causing root and stem rot on luffa and melon (Chikh-Rouhou et al., 2010). The leaves of plants affected with Fusarium, initially exhibit a dull gray green appearance followed by yellowing, wilting and necrosis. A characteristic symptom of Fusarium wilt is discoloration of the vascular system in the vine or stem when viewed in longitudinal or cross section, and this will ultimately lead to the death of the plant (Egel and Martyn, 2007).

*Luffa* spp. are native to Asia (primarily South and Southeast Asia) and are widely cultivated in tropical and subtropical countries (Kumari et al., 2019; Adeyanju et al., 2021). Nine species of *Luffa* have been identified including: *Luffa acutangula*, *L. cylindrica*, *L. aegyptiaca*, *L. operculata*, *L. quinquefida*, *L. saccata*, *L. graveolens, L. astorii* and *L. echinata* (Prakash et al., 2013; Ani et al., 2020). Two cultivated species, *L. acutangula* and *L. aegyptiaca* are widely grown in Asia (Dhillon et al., 2020). Luffa fruit is commonly consumed as a summer vegetable in Asia and Africa (Karmakar et al., 2019). Luffa is a common vegetable in the diets of low-income consumers, containing high content of vitamins, dietary minerals, antioxidant and bioactive phenolic compounds (Dhillon et al., 2020). In most luffa cultivation areas, its production is hampered by Fusarium wilt disease, particularly during the warm seasons. Fusarium wilt has caused significant losses in luffa fields in Taiwan for many years, especially in the farmer selected cultivar ‘White Loofah’ where it causes damage up to 95% under favorable conditions (Lin and Su, 2001). Fusarium wilt is very difficult to control, especially if the use of chemical crop protection agents is not allowed under organic farming. Several strategies have been evaluated for controlling Fusarium wilt in vegetable crops including chemical, physical, and biological methods (Martyn, 2014). Chemical control is often costly and the excessive use of fungicides damages the environment and may harms human health (Lopez Aranda et al., 2016). Although several microbes, including *Bacillus*, *Pseudomonas*, *Trichoderma* and *Penicillium* which have been reported to control Fusarium wilt, do not provide adequate control of this disease under field conditions (Raza et al., 2017; Zhao et al., 2021). Breeding for resistance to Fusarium wilt is the most appropriate, economical, and environmentally promising strategy for controlling this pathogen (Jha et al., 2020). This offers the added advantage to farmers that there is no residue left on the fruits, leading to healthier products for consumers.

Grafting is a common practice in Taiwan for protecting against soil-borne disease and improving fruit yield and quality in cucurbit crops such as bitter gourd (Yetışır et al., 2003). Watermelon grafted on luffa and other cucurbit rootstocks were reported resistant to all races of *F. oxysporum* f. sp. *niveum* (Li et al., 2016). Luffa rootstocks also have been used for protecting grafted cucumber from heat and drought stress, providing optimum root vitality and reducing transpiration rate, photosynthetic inhibition and oxidative stress (Wehner and Ellington, 1997; Liu et al., 2016). There are limited studies on screening *Luffa* spp. germplasm for resistance to *F. oxysporum* f. sp. *luffae*. The objective of this study was to screen the World Vegetable Center genebank (WorldVeg) luffa accessions for resistance to highly aggressive isolates of Fusarium in Taiwan.

## 2 Materials and methods

Experiments were conducted in the Mycology laboratory and greenhouse at WorldVeg HQ in Shanhua, Taiwan during the period January to June, 2019. These comprised 1) inoculation studies and pathogen identification through the use of morphological and molecular markers, 2) screening of 63luffa germplasm accessions and three commercial varieties against a highly aggressive Taiwanese luffa isolate *Fsp-66*, and 3) re-evaluation of fourteen luffa accessions identified from the screening trial as potentially resistant against three aggressive Fusarium isolates collected from luffa, cucumber and bitter gourd. These trials were conducted under temperature-controlled conditions (25-28°C) inside a glass greenhouse (under natural light conditions of approx. 11 hours light and 13 hours dark period).

### 2.1 Pathogenicity test and pathogen identification

Three susceptible commercial cucurbit cultivars: Shimmery luffa, Fountain cucumber, and Moonlight bitter gourd (Known-you Seed Company, Ltd. Taiwan) were included as susceptible checks in the inoculation-pathogenicity tests. To improve seed germination, the luffa seeds were soaked in distilled water for 16 hours at 25°C (Sauer and Burroughs, 1986), followed by ten minutes soaking in 1% sodium hypochlorite solution for surface sterilization (Saleem, 2013), and washed three times with distilled water. The bitter gourd seeds were incubated in a water bath at 52°C for 15 minutes, followed by soaking in distilled water at room temperature for four hours (White et al., 1990), and washed three times with distilled water after soaking in sodium hypochlorite for 10 minutes to prime for uniform germination.

Seven isolates of Fusarium were collected from different locations in Taiwan (**Table 1**) and purified as single-spore cultures. Cultures were grown on PDA (Thermoforma, USA) plates at 28°C for seven to eight days in order to get uniform germination and growth and then used for inoculation or transferred to silica gel for medium to long-term preservation. The isolates were characterized morphologically after 7-8 days growth on PDA plates by measuring the size of 15-30 randomly selected microcondia, macroconidia, conidiophores and assessing for the presence or absence of chlamydospores under the microscope (Olympus, Japan) using T-capture software (Tucsen Photonics Co., Ltd.). Molecular identification to species level was conducted by isolating DNA of each of the isolates separately using FTA^®^ cards (Whatman^®^) followed by PCR amplification of the DNA using the primer pairs ITS-4 (5’-TCC TCC GCT TAT TGA TAT GC-3’)/ITS-5 (5’-GGA AGT AAA AGT CGT AAC AAG G-3’), and Elongation factor-1 (5’-ATG GGT AAG GAR GAC AAG AC-3’)/Elongation factor-2 (5’-GGA RGT ACC AGT SAT CAT GTT-3’) (Altschul et al., 1997). The PCR products were compared by gel electrophoresis (1.6% agarose, 1X TE Buffer at 110 volts for 60 minutes) using GeneDireX^®^ (Taiwan) 100bp DNA ladder as a size marker. The PCR products were also sequenced (Genomics, Taiwan) and the sequences were compared using BLASTn (NCBI database) (Geiser et al., 2004) and the Fusarium-id database (Lin et al., 1996) for species confirmation. Sequences from key isolate *Fsp-66* were deposited in NCBI GenBank. Isolates were also confirmed as being *Fusarium oxysporum* using the species specific primers FOF1/FOR1 (Mishra et al., 2003).

**Table 1:** Morphological characteristics of the Fusarium isolates^a^.

Isolate code	Fusarium species	Location collected	Plant species source	Tissue source	Microconidia size and number	Macroconidia size and number	Conidiophore size and number	Chlamydospores
*Fsp-57*	*F. proliferatum*	Nanjhou, Pingtung,	*L. aegyptiaca*	Seedling	6-10 x 2-3 µ	0	13-20 x 2-3 µ	0
*Fsp-58*	*F. proliferatum*	Nanjhou, Pingtung,	*L. aegyptiaca*	Seedling	7-9 x 2-4 µ	0	11-20 x 2-4 µ	0
*Fsp-66*	*F. oxysporum f.* sp. *luffae*	Liouying, Tainan,	*L. aegyptiaca*	Stem	6-10 x 2-3 µ	25-42 x 3-4 µ	10-15 x 2-3 µ	Present
*Fsp-67*	*F. oxysporum f.* sp. *luffae*	Liouying, Tainan,	*L. aegyptiaca*	Stem	7-10 x 2-4 µ	27-37 x 3-4 µ	9-16 x 2-3 µ	Present
*Fsp-81*	*F. oxysporum f.* sp. *luffae*	Dongshan, Tainan,	*L. aegyptiaca*	Stem	7-10 x 2-3 µ	31-45 x 3-5 µ	9-15 x 2-3 µ	Present
*FoCu-1*	*F.oxysporum f.* sp. *cucumerinum*	National Chung-Hsing University, Taichung City,	*Cucumis sativus*	Stem	8-12 x 3-5 µ	24-38 x 2-4 µ	0	Present
*FoM-6*	*F.oxysporum f.* sp. *momordicae*	Cishan, Kaohsiun,	*Momordica charantia*	Unknown	9-12 x 2-3 µ	23-36 x 3-5 µ	10-13 x 2-3 µ	Present

a These isolates were characterized morphologically after 7-8 days by measuring the size of 15-30 randomly selected microcondia, macroconidia, conidiophores and assessing for the presence or absence of chlamydospores under the microscope.

**Figure 1:**
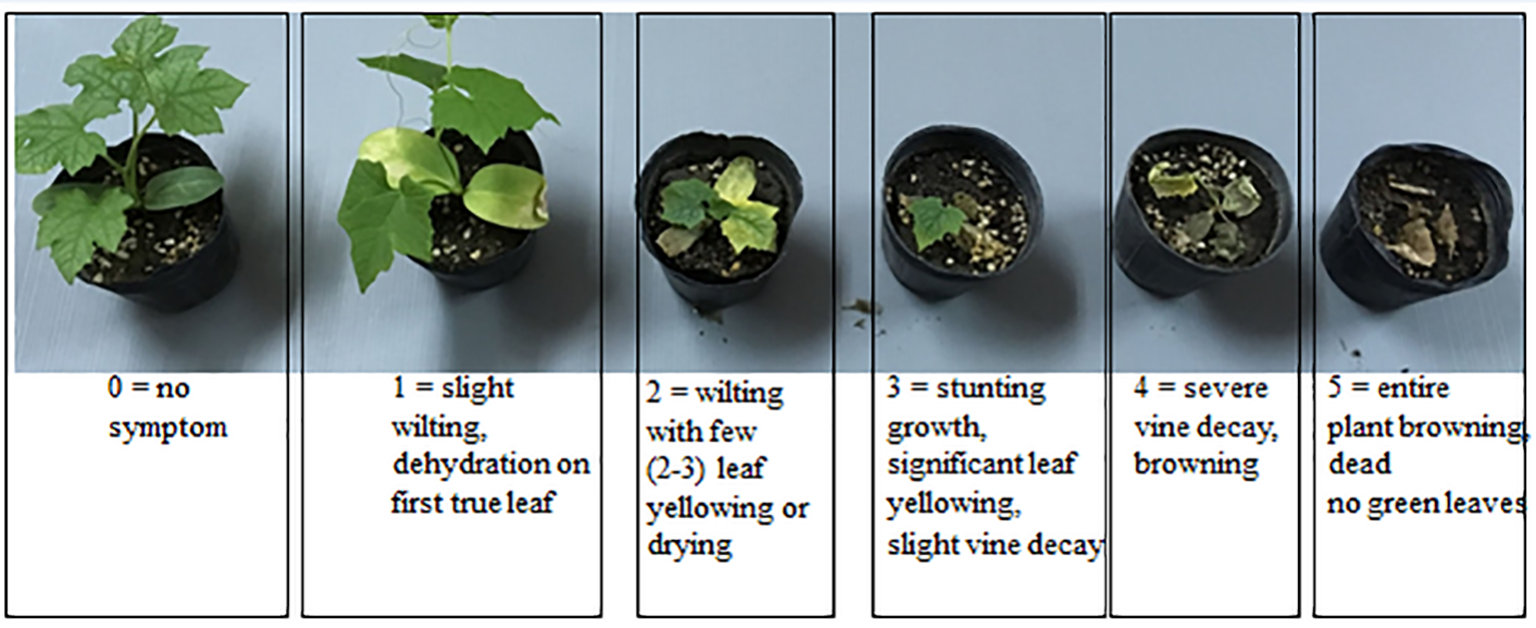
The 0-5 point disease severity rating (DSR) scale used in evaluation trials, with 0 = no symptom; 1 = slight wilting with dehydration of the first true leaf; 2 = wilting with 2-3 leaves yellowing or drying; 3 = stunted growth with significant leaf yellowing and slight vine decay; 4 = severe vine decay with browning; 5 = entire plant brown/dead with no green leaves.

For the initial inoculation/pathogenicity tests, 45 seedlings per each susceptible cucurbit cultivar were arranged in three replications in a randomized complete block design (RCBD) for each Fusarium isolate. Seeds of three susceptible commercial cucurbit cultivars were sown in 72 celled plastic trays (cell size 4.2 x 4.2 x 5 cm^3^) containing sterilized peat moss and the seedlings were watered regularly including once per week with dilute nutrient solution (NPK 2.5%-3.0%-3.0%). The 10 - 15 day-old seedlings were root-prune-inoculated by uprooting them at the 2-3 true leaf stage followed by trimming off one-third of the root tips as modified from (Nash, 1962) and dipping the remaining roots in 150 ml of spore suspension (1 x 10^6^ conidia mL^-1^) for 3 minutes. Each inoculated seedling was transplanted to a separate 3-inch pot containing pasteurized substrate mixture of loamy soil-peat moss-perlite 6:3:1(v:v) and all of the seedlings were maintained under the same conditions as above. Inoculated plants were assessed visually at 7, 14 and 21 days after inoculation, using a 0-5 disease severity rating (DSR) scale where 0 = no symptom; 1 = slight wilting and dehydration on first true leaf; 2 = wilting with two or three leaves yellowing or drying; 3 = stunted growth, significant leaf yellowing, slight vine decay; 4 = severe vine decay and browning; 5 = entire plant brown/dead with no green leaves (**Figure 1**).

### 2.2 Screening all luffa accessions against Fusarium isolate *Fsp-66*

Sixty-three luffa accessions originally collected from different countries [Bangladesh (8), Lao People’s Democratic Republic (35), Cambodia (1), Indonesia (5), USA (1), Philippines (4), Thailand (7) and Vietnam (2)] were obtained from the WorldVeg genebank (**Supplementary Table 1**). Three commercial cultivars (Shimmery, Ajun and Cylinder No. 3) belonging to *L*. *aegyptiaca* were obtained from the Known-you seed co., Ltd.,Taiwan. Commercial cultivar Cylinder No. 3 was used as resistant check while Shimmery cultivar was used as the susceptible check. These resistant and susceptible checks are routinely used in Fusarium screening trials at WorldVeg. Ajun variety was tested for Fusarium resistance in this trial at first time. The WorldVeg genebank accessions and commercial cultivars were evaluated for resistance to an aggressive Taiwanese Fusarium isolate from luffa (*Fsp-66*) using the root pruning inoculation method described above. Seedlings were arranged in a RCBD with three replications and 15 plants per accession and check in each replication.

To confirm infection with Fusarium, seven wilted plants per DSR scale (2,3,4 and 5) were randomly selected for Fusarium isolation on pentachloronitrobenzene (PCNB) media according to the modified method of Plank (1963). Small sections from infected plants (bottom and upper portion of stem) were washed with tap water, cut into discs of about 1 mm in diameter, then dipped in 1% NaClO for 30 sec., followed by washing in distilled water for 30 sec. prior to culturing on PCNB media. All the stem sections on PCNB medium were incubated at 27°C with alternating 12 hours light/dark period (Saint Tein co., ltd.) for uniform germination. Colony morphology was observed after 4-6 days for all the re-isolations.

### 2.3 Re-evaluation trials

Based on the average of DSR scores and extent of segregation in Fusarium resistance within accessions from the initial screening trials, 14 putatively resistant accessions (≤2 DSR) were selected and retested against three isolates (*F. oxysporum* f. sp. *luffae*-66, *F. oxysporum* f. sp. *cucumerinum*-1 and *F. oxysporum* f. sp. *momordicae*-6). Seeds were sown alongside a commercial resistant check (Cylinder No. 3) and a commercial susceptible check (Shimmery) as described above. Seedlings were arranged in a RCBD with three replications and six plants per entry in each replication (i.e., 18 plants per resistant accession and check). 10-15 day-old-plants (2-3 true leaves) were tested as indicated above. Fusarium was re-isolated from three randomly selected with different DSR (2,3,4 and 5) to confirm the pathogen.

### 2.4 Statistical analysis

Mean DSR was calculated based on the DSR rating on 21 days after inoculation for each accession. Statistical analyses were conducted using the statistical software SAS (version 9.1; SAS Institute, Cary, NC). Data of DSR were subjected to one-way analysis of variance (ANOVA), and mean separations were determined using the Tukey-Kramer honestly significant difference (HSD) test (*P* = 0.05). Percentage of resistant plants was calculated as follows: number of resistant seedlings (DSR ≤ 2)/total number of seedlings for each treatment. The accessions were categorized for resistance based on mean DSR values as resistant (DSR ≤2), moderately susceptible (DSR 2.1 to 3), susceptible (DSR 3.1 to <4.9) and highly susceptible (DSR =5).

## 3 Results

### 3.1 Pathogen identification

Two isolates of Fusarium from luffa (Fsp-57 and Fsp-58) were identified as being *F. proliferatum* species by the microscopy morphological study and ITS sequencing, while the other three luffa isolates, one cucumber and one bitter gourd isolate were identified as *F. oxysporum* (**Table 1**). Sequences from key isolate Fsp-66 were deposited in NCBI GenBank as accessions OQ381099 (ITS) and OQ407676 (EF). Some of the morphological features (macro and microconidia, conidiophore and presence/absence of chlamydospores) for the three isolates are presented in the **Supplementary Figure 1**.

### 3.2 Pathogenicity test

**Figure 2:**
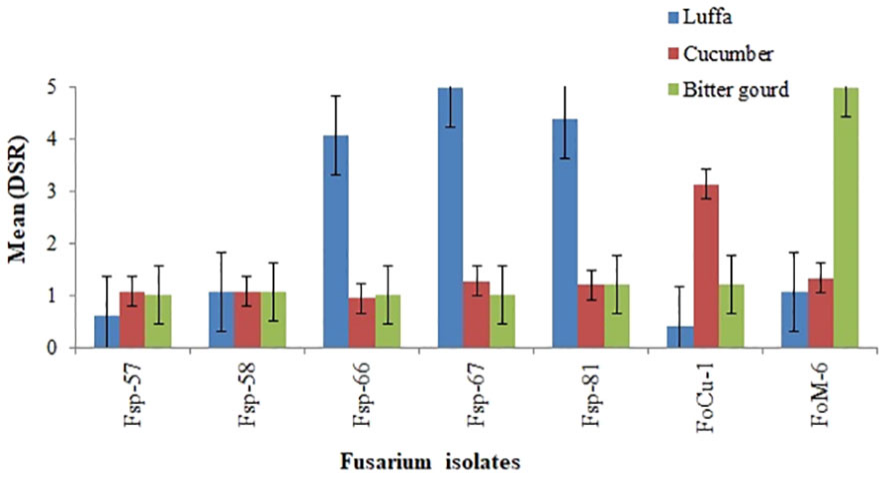
Mean disease severity ratings (DSR) values at 21 days after inoculation for luffa (Shimmery cv.), cucumber (Fountain cv.) and bitter gourd (Moonlight cv.) when challenged with Fusarium isolates from luffa (*Fsp*-57, *Fsp* -58, *Fsp*-66, *Fsp*-67 and *Fsp*-81), from cucumber (*FoC*-1), and from bitter gourd (*Fom*-6).

The mean disease severity ratings (DSR) values for the seven Fusarium isolates at 21 days after inoculation (dai) on commercial susceptible luffa (Shimmery cv.), cucumber (Fountain cv.) and bitter gourd (Moonlight cv.), are presented in **Figure 2**. The mean DSR results 21 dai showed that the three luffa isolates *Fsp-66*, *F*sp-67 and *F*sp-81 were caused high disease severity in the susceptible luffa (Shimmery cv.) with DSR in the range of 4-5. This cultivar was used as susceptible check in the screening and re-evaluation trials. Two more isolates (*Foc*-1 and *Fom*-6) caused high disease severity (3-5 DSR) in the cucumber and bitter gourd susceptible varieties. However, the luffa isolates *F*p-57 and *Fp*-58 caused low disease severity in the luffa, cucumber and bitter gourd cultivars. Other cucumber and bitter gourd isolates of Fusarium (*Fc*-1 and *F. oxysporum* f. sp. *momordicae*-6) did not cause high disease severity in the luffa cultivars.

### 3.3 Screening luffa accessions against Fusarium isolate *Fsp-66*

There were highly significant differences (*P<0.05*) among luffa accessions and checks for DSR. Results revealed that the mean DSR value ranged from 0.53 for VI055596 to 5 for accession VI054859 in 63 genebank accessions (**Table 2**). Commercial resistant check ‘Cylinder No. 3’ displayed the lowest mean DSR 0.27 with 100% resistance percentage. In contrast, commercial variety ‘Shimmery’ which was used as susceptible check showed a mean DSR of 4.07 and the resistance percentage of 26.67% as expected. Out of 63 genebank accessions, 25 accessions were categorized as resistant with mean DSR value (≤2), 13 as moderately susceptible (2.1 to ≤3), 24 as susceptible (3.1 to <5) and one as highly susceptible (=5). Three randomly selected wilted plants per accession and check with different DSR (2, 3, 4 and 5) confirmed infection with Fusarium when their tissues were isolated on PCNB media.

**Table 2:** Evaluation of luffa accessions against an aggressive isolate of *Fusarium oxysporum* f. sp. *luffae (Fsp*-66) 21 days after inoculation.

Luffa species	WorldVeg accession code	Mean DSR ± S.E. ^a^	Resistance %^b^	Resistance category ^c^
*L. aegyptiaca*	Cylinder No. 3^d^	0.27 ± 0.18 a1	100.00	R
*L. aegyptiaca*	VI056199	0.53 ± 0.07 za1	100.00	R
*Luffa* sp.	VI057235	0.53 ± 0.07 za1	100.00	R
*L. aegyptiaca*	VI055596	0.53 ± 0.18 za1	100.00	R
*L. acutangula*	VI055376	0.87 ± 0.13 y-a1	100.00	R
*L. acutangula*	VI055375	0.91 ± 0.07 x-a1	100.00	R
*L. aegyptiaca*	VI055950-C	0.93 ± 0.07 w-a1	100.00	R
*L. acutangula*	VI055373-B	0.93 ± 0.07 w-a1	100.00	R
*L. aegyptiaca*	VI055857	0.93 ± 0.18 w-a1	100.00	R
*L. aegyptiaca*	VI055950-A	1.00 ± 0.00 v-a1	100.00	R
*L. aegyptiaca*	Ajun	1.00 ± 0.00 v-a1	100.00	R
*L. aegyptiaca*	VI055950-B	1.00 ± 0.07 v-a1	100.00	R
*L. acutangula*	VI055943	1.11 ± 0.10 v-a1	100.00	R
*L. aegyptiaca*	VI038112	1.10 ± 0.07 u-a1	100.00	R
*L. acutangula*	VI055749	1.27 ± 0.18 u-z	100.00	R
*L. aegyptiaca*	VI055955-A	1.33 ± 0.33 y-z	93.33	R
*L. aegyptiaca*	VI055716	1.40 ± 0.42 y-z	85.71	R
*L. aegyptiaca*	VI055688	1.47 ± 0.24 t-z	86.67	R
*L. aegyptiaca*	VI047391	1.53 ± 0.23 s-z	100.00	R
*L. aegyptiaca*	VI055652	1.60 ± 0.58 r-y	80.00	R
*L. acutangula*	VI055374	1.67 ± 0.31 r-y	86.67	R
*L. aegyptiaca*	VI055955-C	1.67 ± 0.24 q-y	86.67	R
*L. aegyptiaca*	VI055949	1.80 ± 0.46 q-y	80.00	R
*L. acutangula*	VI039833	1.87 ± 0.13 p-y	100.00	R
*L. acutangula*	VI055372	1.87 ± 0.47 p-y	91.67	R
*L. aegyptiaca*	VI055705	1.93 ± 0.57 o-x	73.33	R
*L. aegyptiaca*	VI055802	2.00 ± 0.61 n-w	69.23	R
*L. acutangula*	VI055373-A	2.03 ± 0.39 n-v	91.67	MS
*Luffa* sp.	VI043135	2.20 ± 0.46 n-u	80.00	MS
*L. acutangula*	VI055377	2.27 ± 0.18 n-u	73.33	MS
*L. aegyptiaca*	VI055979	2.27 ± 0.37 n-u	69.23	MS
*L. acutangula*	VI038108	2.47 ± 0.24 m-t	73.33	MS
*L. aegyptiaca*	VI055837	2.53 ± 0.85 m-s	60.00	MS
*L. acutangula*	VI039803	2.60 ± 0.59 l-s	75.00	MS
*Luffa* sp.	VI043136	2.60 ± 0.12 l-r	73.33	MS
*L. acutangula*	VI038107	2.70 ± 0.10 k-q	75.00	MS
*Luffa* sp.	VI043137	2.87 ± 0.55 j-p	60.00	MS
*L. aegyptiaca*	VI055955-B	2.87 ± 0.71 j-p	53.33	MS
*Luffa* sp.	VI047405	2.93 ± 0.47 i-o	60.00	MS
*L. acutangula*	VI040045	3.00 ± 0.35 h-n	53.33	MS
*Luffa* sp.	VI043139	3.33 ± 0.24 g-m	53.33	S
*L. acutangula*	VI039813	3.33 ± 0.24 g-m	46.67	S
*L. aegyptiaca*	VI038104	3.40 ± 0.21 g-m	45.45	S
*L. acutangula*	VI040961	3.47 ± 0.47 f-m	40.00	S
*L. acutangula*	VI038105	3.60 ± 0.35 f-l	40.00	S
*L. aegyptiaca*	VI055686	3.67 ± 0.44 e-k	33.33	S
*L. aegyptiaca*	VI055978	3.67 ± 0.71 e-k	33.33	S
*L. aegyptiaca*	VI034708	3.77 ± 0.62 d-j	33.33	S
*L. aegyptiaca*	VI055967	3.80 ± 0.20 d-j	33.33	S
*L. aegyptiaca*	VI055726	3.80 ± 0.61 d-j	26.67	S
*L. aegyptiaca*	VI054856	3.87 ± 0.30 c-j	30.00	S
*L. aegyptiaca*	VI055691	3.93 ± 0.71 b-i	26.67	S
*L. aegyptiaca*	VI055930	4.00 ± 0.31 a-h	26.67	S
*Luffa* sp.	VI043138	4.06 ± 0.13 a-g	26.67	S
*L. aegyptiaca*	Shimmery^d^	4.06 ± 0.48 a-g	26.67	S
*L. acutangula*	VI046065	4.13 ± 0.43 a-g	22.22	S
*L. aegyptiaca*	VI055829	4.20 ± 0.46 a-g	20.00	S
*L. aegyptiaca*	VI055831	4.27 ± 0.47 a-g	20.00	S
*L. aegyptiaca*	VI055869	4.47 ± 0.29 a-f	13.33	S
*L. aegyptiaca*	VI055865	4.47 ± 0.44 a-f	6.67	S
*L. aegyptiaca*	VI047205	4.67 ± 0.33 a-e	8.33	S
*L. aegyptiaca*	VI055994	4.73 ± 0.27 a-d	6.67	S
*L. aegyptiaca*	VI055867	4.80 ± 0.20 a-d	6.67	S
*L. aegyptiaca*	VI055658	4.87 ± 0.13 abc	0.00	S
*L. aegyptiaca*	VI055693	4.93 ± 0.07 ab	0.00	S
*L. aegyptiaca*	VI054859	5.00 ± 0.00 a	0.00	HS

a Means ± SE (n = 45) followed by different letters are significantly different according to Tukey-Kramer honestly significant difference (HSD) test (P<0.05). Data were statistically analyzed using one-way analysis of variance (ANOVA).

b Percentage resistant plants = number of resistant seedlings (DSR ≤ 2)/total number of seedlings for each treatment.

c HS, Highly susceptible; S, Susceptible; MS, Moderately susceptible; R, Resistant.

d Cylinder No. 3 and Shimmery were used as resistant and susceptible checks, respectively.

Accessions are arranged in ascending order of their mean disease severity ratings (DSR).

### 3.4 Re-evaluation trial

Fourteen genebank luffa accessions previously identified as resistant (≤2 DSR) and exhibited no segregation in Fusarium resistance within accessions in screening trials were tested against three Fusarium isolates from luffa (*Fsp-66*), cucumber (*FoCu-1*), and bitter gourd (FoM-6). The commercial resistant check cultivar, Cylinder No. 3, and susceptible check cultivar, Shimmery, were also included in the test. In screening with *Fsp-66*, eleven accessions and the resistant check were confirmed as resistant based on their mean DSR values (DSR ≤2), whereas three accessions were categorized as moderately susceptible (DSR 2.1 to ≤3), and only the susceptible check (Shimmery cv.) was confirmed as susceptible (DSR 3.1 to <5) (**Table 3**). Fungal isolation from tissue of three randomly selected wilted plants per accession and check on PCNB media confirmed infection with Fusarium. Fusarium was rarely isolated from plants showing a DSR of 2 or less.

**Table 3:** Evaluation of selected luffa accessions against three *Fusarium oxysporum* isolates (*F. oxysporum* f. sp. *luffae*-66, *F. oxysporum* f. sp. *cucumerinum*-1 and *F. oxysporum* f. sp. *momordicae*-6) 21 days after inoculation.

Luffa species	WorldVeg accession code	Mean DSR ± S.E. ^a^	Resistance % ^b^	Resistance category ^c^
F. oxysporum f. sp. luffae-66				
*L. aegyptiaca*	Cylinder No. 3^d^	0.84 ± 0.17 f	100.00	R
*L. acutangula*	VI055373-B	1.00 ± 0.00 ef	100.00	R
*L. aegyptiaca*	VI038112	1.06 ± 0.06 ef	100.00	R
*L. aegyptiaca*	VI047391	1.11 ± 0.06 def	94.44	R
*L. acutangula*	VI055375	1.13 ± 0.07 def	100.00	R
*L. acutangula*	VI055372	1.23 ± 0.15 def	93.75	R
*Luffa* sp.	VI057235	1.30 ± 0.07 def	82.35	R
*L. aegyptiaca*	VI055596	1.42 ± 0.42 c-f	83.33	R
*L. acutangula*	VI055376	1.58 ± 0.30 c-f	83.33	R
*L. aegyptiaca*	VI056199	1.71 ± 0.20 cde	70.59	R
*L. aegyptiaca*	VI055950-B	1.77 ± 0.15 cde	76.47	R
*L. acutangula*	VI039833	1.89 ± 0.24 bcd	77.78	R
*L. aegyptiaca*	VI055950-A	2.22 ± 0.62 bc	61.11	MS
*L. aegyptiaca*	VI055950-C	2.22 ± 0.62 bc	50.00	MS
*L. aegyptiaca*	VI055857	2.67 ± 0.19 b	55.56	MS
*L. aegyptiaca*	Shimmery^d^	4.78 ± 0.22 a	5.56	S
F. oxysporum f. sp. cucumerinum-1				
*L. aegyptiaca*	Cylinder No. 3^d^	0.67 ± 0.10 c	100.00	R
*L. aegyptiaca*	VI056199	1.05 ± 0.15 bc	94.44	R
*L. acutangula*	VI055373-B	1.05 ± 0.15 bc	94.44	R
*Luffa* sp.	VI057235	1.05 ± 0.15 bc	94.44	R
*L. acutangula*	VI039833	1.17 ± 0.17 bc	90.00	R
*L. acutangula*	VI055376	1.39 ± 0.24 bc	88.89	R
*L. aegyptiaca*	VI055950-B	1.56 ± 0.40 ab	72.22	R
*L. aegyptiaca*	VI047391	1.61 ± 0.39 ab	72.22	R
*L. aegyptiaca*	VI055596	1.67 ± 0.19 ab	66.67	R
*L. aegyptiaca*	VI055950-C	1.67 ± 0.26 ab	66.67	R
*L. aegyptiaca*	VI055950-A	1.67 ± 0.35 ab	77.78	R
*L. aegyptiaca*	Shimmery^d^	1.72 ± 0.50 ab	66.67	R
*L. aegyptiaca*	VI055857	2.33 ± 0.19 a	33.33	MS
F. oxysporum f. sp. momordicae-6				
*L. aegyptiaca*	VI056199	0.95 ± 0.15 f	94.44	R
*L. aegyptiaca*	Cylinder No. 3^d^	1.00 ± 0.25 ef	88.89	R
*L. aegyptiaca*	VI047391	1.06 ± 0.11 def	100.00	R
*L. aegyptiaca*	VI055950-C	1.06 ± 0.20 def	88.89	R
*L. acutangula*	VI055376	1.30 ± 0.15 c-f	84.62	R
*L. aegyptiaca*	VI055950-B	1.43 ± 0.11 b-f	77.78	R
*L. aegyptiaca*	VI055596	1.50 ± 0.10 b-f	77.78	R
*L. acutangula*	VI039833	1.69 ± 0.39 b-e	70.00	R
*Luffa* sp.	VI057235	1.72 ± 0.15 a-d	72.22	R
*L. aegyptiaca*	VI055857	1.78 ± 0.40 abc	61.11	R
*L. aegyptiaca*	VI055950-A	1.79 ± 0.40 abc	64.71	R
*L. aegyptiaca*	Shimmery^d^	2.11 ± 0.29 ab	44.44	MS
*L. acutangula*	VI055373-B	2.42 ± 0.09 a	41.18	MS

a Means ± SE (n = 18) followed by different letters are significantly different according to Tukey-Kramer honestly significant difference (HSD) test (P<0.05). Data were statistically analyzed using one-way analysis of variance (ANOVA).

b Percentage resistant plants = number of resistant seedlings (DSR ≤ 2)/total number of seedlings for each treatment.

c HS, Highly susceptible; S, Susceptible; MS, Moderately susceptible; R, Resistant.

d Cylinder No. 3 and Shimmery were used as resistant and susceptible checks, respectively.

Accessions are arranged in ascending order of their mean disease severity ratings (DSR).

Only 11 of the resistant luffa accessions from re-evaluation trial had sufficiently high germination to be included in the screening with *FoC* 1, and of these, ten accessions were categorized as resistant (mean DSR ≤ 2), while one (VI055857) was moderately susceptible (mean DSR 2.1 to ≤3) (**Table 3**). Both the Cylinder No. 3 (resistant check) and Shimmery (susceptible check) were categorized as resistant (mean DSR ≤2). When stem sections from three randomly selected wilted plants of the moderately susceptible accession (VI055857) with different DSRs (3 and 5) were plated on PCNB media, Fusarium was re-isolated.

As with FoC-1, only 11 of the luffa accessions were available for the screening with *FoM*-6. Of these, 10 were categorized as resistant (mean DSR ≤2) and one (VI055373-B) was moderately susceptible (mean DSR 2 ≤ 3) (**Table 3**). The resistant check (Cylinder No. 3 cv.) exhibited resistance to isolate FoM-6 (mean DSR ≤2), while the susceptible check (Shimmery cv.) was moderately susceptible (mean DSR ≤3).

## 4 Discussion

*Formae speciales* of *F. oxysporum* typically have a narrow host range, often restricted to a single plant species (Kawai et al., 1958; Kistler et al., 1998; Van Dam et al., 2017). One of the Fusarium isolates from luffa (*Fsp.* -66) was assessed to be aggressive based on the high mean DSR attained when inoculated on the susceptible luffa (Shimmery cv.). This is in agreement with previous work on this isolate at WorldVeg, which also showed it to have a low level of cross pathogenicity to other hosts confirming its host specific nature. These findings were also supported by the work of other groups (Lin and Su, 2001; Asma et al., 2018; Borah et al., 2018). The Fusarium isolates from cucumber and bitter gourd also showed a high level of host specificity and a low level of cross pathogenicity to other hosts. Many plant-pathogenic fungi are highly host-specific and interactions between them and their host evolved at the time of speciation of the respective host plants (Chehri et al., 2011). The isolates of *F. proliferatum* isolated from luffa caused only mild disease on luffa, cucumber and bitter gourd and were regarded as only weakly pathogenic on these hosts. From the pathogenicity test and in agreement with other studies, *formae speciales* of *F. oxysporum* were found to be the major cause of root and stem rot (wilt) of cucurbit plants, with*. F*. *proliferatum, F*. *equiseti, F*. *semitectum* and *F*. *solani* as only occasional or minor causes (Barnett and Hunter, 1972).

All the isolates tested in this study had the morphological features of pathogenic Fusarium species (Gilman, 1957; Booth, 1971; Namiki et al., 1994; Edel-Hermann and LeComte, 2019). The molecular analysis using Internal Transcribed Spacer (ITS) and Elongation factor (EF) sequences confirmed the isolates with greatest pathogenicity were *F. oxysporum* (Chen et al., 2019), while the two isolates with weaker pathogenicity on cucurbit species were identified as *F. proliferatum*.

Most accessions identified as resistant to Fusarium wilt in the initial screening trial showed a low segregation rate (91 to 100% resistance) within themselves and will probably need minimal breeding effort to purify and fix the resistance. On the other hand, the genebank accessions categorized as moderately susceptible showed more segregation (53 to 91% resistance) within themselves, indicating that it may be possible to select and fix higher levels of resistance from individual plants from these accessions. These accessions might also have good horticultural traits such as earliness; increase the percentage of pistillate flowers and fruit quality which could be targeted by breeders. The accessions found to be susceptible or highly susceptible to Fusarium wilt are unsuitable for selection for resistance although they may have other desired horticultural characteristics (Jeger and Viljanen-Rollinson, 2001).

Accessions identified as resistant in the initial screening trial with segregation (69 - 86% resistance) will be selfed and selected to fix the resistance. Although the commercial luffa resistant check (Cylinder No. 3 cv.) was more resistant than the genebank accessions, the accessions may carry different resistance genes that are suitable for breeding and possibly combining with that in Cylinder No. 3, to provide effective and durable resistance to manage this serious disease. The commercial susceptible check (Shimmery cv.) showed the expected reaction to Fusarium and was recommended for use as susceptible control for Luffa Fusarium strains in future WorldVeg experiments. The higher final (21 dai) DSR observed in some accessions may be explained by a slower initial rate of disease development (Duncan and Waller, 1969 Shetty and Wehner, 2002).

Only the accessions categorized as resistant and showing low levels of segregation (91-100%) were selected for re-evaluation trials. Resistance was confirmed in all 11 accessions the re-evaluation trials.

In re-evaluation screening, the Fusarium isolates from cucumber and bitter gourd showed a low level of cross pathogenicity to most of the luffa accessions, and only two accessions showed moderate susceptibility to these isolates. Resistant luffa accessions identified in the present study were also resistant to Fusarium isolates of cucumber and bitter gourd, indicating their usefulness for using as rootstocks to these crops to manage this pathogen. These results were similar to those of Williams (1996).

The root-trimming plus dipping in spore suspension was a reliable method for screening cucurbit accessions for reaction to different Fusarium isolates. *Fusarium oxysporum formae speciales* isolates from other cucurbit species were only weakly cross-pathogenic to luffa, with susceptible check Shimmery exhibiting a moderately susceptible reactions (mean DSR 2-3). Based on the results, the aggressive isolate, *Fsp-66* was identified as highly suitable for screening luffa accessions for resistance to Fusarium wilt. The accessions that showed resistance to *Fsp-66* are potentially useful both for incorporation into a luffa breeding program, and also for use as Fusarium wilt resistant rootstock for grafting with other cucurbit varieties as scions. However, these accessions first have to be re-tested, including in open field to known to be uniformly infested with high levels of pathogenic isolates *Fusarium oxysporum f.* spp.and in different geographic locations and agro-environments, and if necessary, fix the resistance through selfing and selection. At that point it would be necessary to determine if the resistances in the different accessions are allelic and how the resistance functions. Since root knot nematodes (RKN, *Meloidogyne* spp.) are another soil-borne root disease of cucurbits including luffa (Ayala-Donas et al., 2020), it would also be useful to determine if any of the Fusarium wilt resistant luffa accessions are also resistant to common RKN species or strains, or if it will be necessary to combine RKN and Fusarium wilt resistances from different sources. A potential interaction between nematode and Fusarium was reported in cucurbits under greenhouse condition. Sumner and Johnson (1973) found that incidence of Fusarium wilt increased significantly in soils infested with root knot nematode than in soils not infested or soils with very low infestation. However, no differences in incidence of Fusarium wilt were observed when watermelon or interspecific hybrid squash was inoculated with Fusarium and nematode (Seo and Kim, 2017; Keinath and Agudelo, 2018). Luffa rootstock that is resistant to both Fusarium wilt and nematode would offer greater benefits to farmers through minimizing the use of chemicals and improve yield and fruit quality of cucurbit varieties sensitive to soil-borne diseases.

## 5 Conclusions

Cucurbits are an important class of vegetables which are cultivated around the globe, but Fusarium wilt is a major disease in cucurbits and accounts for high production and economic losses worldwide. However, it may not be as susceptible to such a range of *formae speciales* or strains of Fusarium as most other cucurbit species and so may have the potential to be used as rootstocks for other cucurbits. In this context, this study was conducted in systematically arranged experiments with the objectives of 1) identifying the most aggressive Fusarium isolate available from luffa, 2) confirming its species identification by morphological and molecular means, 3) using that isolate in screening to identify potentially resistant luffa accessions and 4) confirming the resistance in those accessions by screening with the aggressive isolate and two other Fusarium isolates from other cucurbit species. The most aggressive isolate was identified as *Fusarium oxysporum f.sp. luffae*, evaluation of 63 luffa accessions against this isolate, and 14 of 63 luffa accessions from the WorldVeg gene bank were identified as highly resistant to Fusarium wilt. Three of the 14 resistant accessions were moderately susceptible to the aggressive isolate on rescreening, and similarly although most were resistant when screened with *F. oxysporum f.sp cucumerinum* from cucumber and *F. oxysporum f.sp. momordicae* from bitter gourd, there were two moderately susceptible to these other isolates. This is the first report of Fusarium wilt resistance in Luffa and these sources will be valuable for Luffa breeding for development of Fusarium-resistant varieties/rootstocks.

## Data availability statement

The original contributions presented in the study are included in the article/**Supplementary Material**. Further inquiries can be directed to the corresponding author.

## Author contributions

Conceptualization, MR, SB and Z-MS. Methodology, MR, SB and Z-MS. Software, MR, SB and Z-MS. Validation, MR, SB, LK and Z-MS. Formal analysis, DT and SB. Investigation, MR, SB and Z-MS. Resources, MR. Data curation MR, SB, DT, Z-MS. Writing—original draft preparation SB and MR. Writing—review and editing, MR, DT and LK. Visualization, MR, SB and Z-MS. supervision, MR and LK Project administration, MR. Funding acquisition, MR All authors contributed to the article and approved the submitted version.
