# Supplementary material for: Novel sources of resistance to fusarium wilt in Luffa species
Source: Front Plant Sci. 2023 Jun 9;14:1116006. doi: 10.3389/fpls.2023.1116006 (PMC10288365; doi:10.3389/fpls.2023.1116006)
Supplement: Supplementary file 1 [file Image_1.pdf]

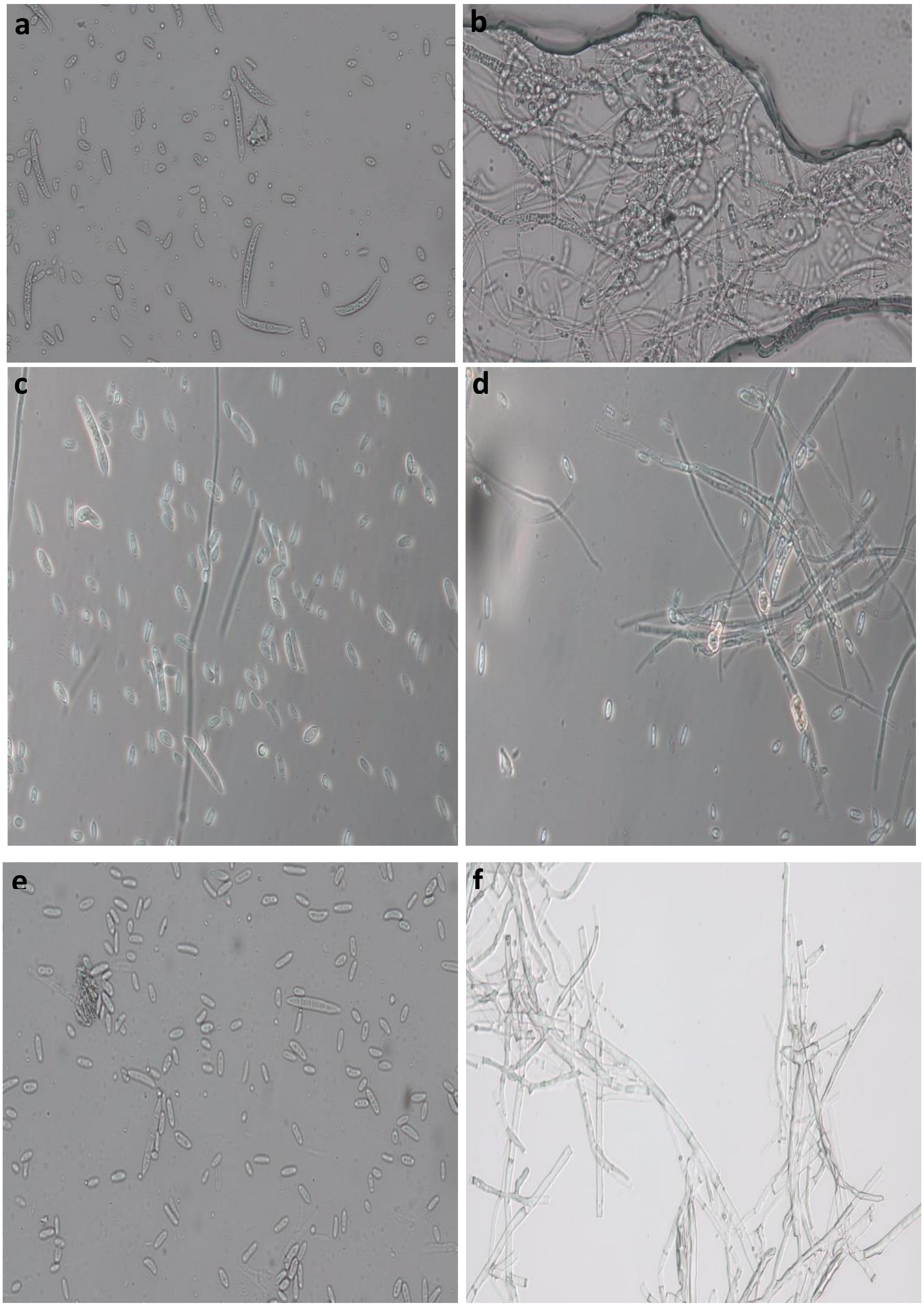

Supplementary Figure 1. *Fusarium oxysporum* f. sp. *luffae*-66 macro & microconidia (a) chlamydospores (b), *Fusarium oxysporum* f. sp. *cucumerinum*-1 macro & microconidia (c) chlamydospores (d), *Fusarium oxysporum* f. sp. *momordicae*-6 macro & microconidia (e) conidiophore (f).
