# Supplementary material for: Novel sources of resistance to fusarium wilt in Luffa species
Source: Front Plant Sci. 2023 Jun 9;14:1116006. doi: 10.3389/fpls.2023.1116006 (PMC10288365; doi:10.3389/fpls.2023.1116006)
Supplement: Supplementary file 2 [file Table_1.pdf]

Supplementary Table 1: Luffa accessions used for evaluation for resistance to fusarium wilt

Number	Species	WorldVeg accession code	Origin of accession
1	*Luffa aegyptiaca*	VI054859	Lao People's Democratic Republic
2	*L. aegyptiaca*	VI047205	Lao People's Democratic Republic
3	*L. acutangula*	VI046065	United States of America
4	*Luffa* sp.	VI043138	Indonesia
5	*L. aegyptiaca*	VI054856	Lao PDR
6	*L. aegyptiaca*	VI034708	Philippines
7	*L. acutangula*	VI038105	Thailand
8	*L. acutangula*	VI040961	Thailand
9	*L. aegyptiaca*	VI038104	Thailand
10	*L. acutangula*	VI039813	Philippines
11	*Luffa* sp.	VI043139	Indonesia
12	*L. acutangula*	VI040045	Thailand
13	*Luffa* sp.	VI047405	Viet Nam
14	*Luffa* sp.	VI043137	Indonesia
15	*L. acutangula*	VI038107	Thailand
16	*L. acutangula*	VI039803	Philippines
17	*Luffa* sp.	VI043136	Indonesia
18	*L. acutangula*	VI038108	Thailand
19	*Luffa* sp.	VI043135	Indonesia
20	*L. acutangula*	VI055373-A	Bangladesh
21	*L. acutangula*	VI055372	Bangladesh
22	*L. acutangula*	VI039833	Philippines
23	*L. aegyptiaca*	VI047391	Viet Nam
24	*L. aegyptiaca*	VI038112	Thailand
25	*L. aegyptiaca*	VI055693	Lao People's Democratic Republic
26	*L. aegyptiaca*	VI055658	Lao People's Democratic Republic
27	*L. aegyptiaca*	VI055867	Lao People's Democratic Republic
28	*L. aegyptiaca*	VI055994	Lao People's Democratic Republic
29	*L. aegyptiaca*	VI055869	Lao People's Democratic Republic

30	*L. aegyptiaca*	VI055865	Lao People's Democratic Republic
31	*L. aegyptiaca*	VI055831	Lao People's Democratic Republic
32	*L. aegyptiaca*	VI055829	Lao People's Democratic Republic
33	*L. aegyptiaca*	VI055930	Lao People's Democratic Republic
34	*L. aegyptiaca*	VI055691	Lao People's Democratic Republic
35	*L. aegyptiaca*	VI055726	Lao People's Democratic Republic
36	*L. aegyptiaca*	VI055967	Lao People's Democratic Republic
37	*L. aegyptiaca*	VI055686	Lao People's Democratic Republic
38	*L. aegyptiaca*	VI055978	Lao People's Democratic Republic
39	*L. aegyptiaca*	VI055955-B	Lao PDR
40	*L. aegyptiaca*	VI055837	Lao PDR
41	*L. acutangula*	VI055377	Bangladesh
42	*L. aegyptiaca*	VI055979	Lao PDR
43	*L. aegyptiaca*	VI055802	Lao PDR
44	*L. aegyptiaca*	VI055705	Lao PDR
45	*L. aegyptiaca*	VI055949	Lao PDR
46	*L. aegyptiaca*	VI055955-C	Lao PDR
47	*L. acutangula*	VI055374	Bangladesh
48	*L. aegyptiaca*	VI055652	Lao PDR
49	*L. aegyptiaca*	VI055688	Lao PDR
50	*L. aegyptiaca*	VI055716	Lao PDR
51	*L. aegyptiaca*	VI055955-A	Lao PDR
52	*L. acutangula*	VI055749	Lao PDR
53	*L. acutangula*	VI055943	Lao PDR
54	*L. aegyptiaca*	VI055950-B	Lao PDR
55	*L. aegyptiaca*	VI055950-A	Lao PDR
56	*L. aegyptiaca*	VI055950-C	Lao PDR
57	*L. acutangula*	VI055375	Bangladesh
58	*L. aegyptiaca*	VI055857	Lao PDR
59	*L. acutangula*	VI055373-B	Bangladesh
60	*L. acutangula*	VI055376	Bangladesh
61	*L. aegyptiaca*	VI055596	Lao PDR
62	*L. aegyptiaca*	VI056199	Bangladesh

63	*Luffa* sp.	VI057235	Cambodia
